# The theory of thermoelasticity with a memory-dependent dynamic response for a thermo-piezoelectric functionally graded rotating rod

**DOI:** 10.1038/s41598-023-36371-2

**Published:** 2023-06-03

**Authors:** Ahmed E. Abouelregal, S. S. Askar, M. Marin, Badahiould Mohamed

**Affiliations:** 1 Department of Mathematics, Faculty of Science, Mansoura University, Mansoura, 35516 Egypt; 2 Department of Statistics and Operations Research, College of Science, King Saud University, P.O. Box 2455, Riyadh, 11451 Saudi Arabia; 3 Department of Mathematics and Computer Science, Transilvania University of Brasov, Brasov, Romania; 4 Faculty of Sciences and Technology, University of Nouakchott, Nouakchott, Mauritania

**Keywords:** Materials science, Mathematics and computing

## Abstract

By laminating piezoelectric and flexible materials during the manufacturing process, we can improve the performance of electronic devices. In smart structure design, it is also important to understand how the functionally graded piezoelectric (FGP) structure changes over time when thermoelasticity is assumed. This is because these structures are often exposed to both moving and still heat sources during many manufacturing processes. Therefore, it is necessary to conduct theoretical and experimental studies of the electrical and mechanical characteristics of multilayer piezoelectric materials when they are subjected to electromechanical loads and heat sources. Since the infinite speed of heat wave propagation is a challenge that classical thermoelasticity cannot address, other models based on extended thermoelasticity have been introduced. For this reason, the effects of an axial heat supply on the thermomechanical behavior of an FGP rod using a modified Lord-Shulman model with the concept of a memory-dependent derivative (MDD) will be explored in this study. The exponential change of physical properties in the direction of the axis of the flexible rod will be taken into account. It was also assumed that there is no electric potential between the two ends of the rod while it is fixed at both ends and thermally isolated. Applying the Laplace transform method, the distributions of the physical fields under investigation were calculated. The obtained results were compared to those in the corresponding literature with varying heterogeneity values, kernel functions, delay times, and heat supply speeds. It was discovered that the studied physical fields and the dynamic behavior of the electric potential are weakened by increasing the inhomogeneity index.

## Introduction

Traditional models of thermoelasticity, which assume that heat pulses can move at an infinite rate, are at odds with many scientific observations. In order to explain this discrepancy, researchers have spent the past five decades developing non-classical models that explain the limited speed at which heat can be transferred through elastic solids. Experiments that show the second sound influence, which is a wave-like speed of heat transfer in materials, back up generalized systems of thermoelasticity that use the hyperbolic-type heat transport equation instead of the parabolic-type equation used in the traditional coupled thermoelastic framework.

Lord and Shulman^1^ discussed expanding Fourier's law of heat transfer by including a flux-rate term and developing a general form that includes a hyperbolic-type heat conduction equation with a limited thermal pulse speed. This would make for a better thermoelasticity framework. Green and Lindsay^2^ used relaxation time ideas that don't go against the standard Fourier law of heat transfer to build a thermoelastic framework that predicts a heat transfer speed limit based on the temperature change rate. Further development of thermoelasticity was made by Green and Naghdi^3–5^, who introduced the so-called first, second, and third models of extended thermoelasticity of homogeneous elastic materials. The mathematical formulation of the first model (GN-I) can be reduced to the classical Fourier concept of heat transfer. In contrast, the second and third theories (GN-II and GN-III) allow thermal waves to move at restricted speeds.

Several recent articles show that there is more and more interest in the field of electro-elasticity, which is the area of piezoelectric materials where the mechanical and electric areas are linked. There are a variety of smart materials and systems, some of which include sensors, actuators, electrical and mechanical transducers, microgenerators, and ultrasonic biomechanical optoelectronic elements that make use of piezoelectric composite materials due to their high sensitivity and low mechanical losses. Most people concur that piezoelectric composite—made by combining piezoelectric ceramic fibers (PZT) with a matrix—works best with ultrasonics. PZT fibers that are mixed into an epoxy matrix make great active fiber composites for building aerospace vehicles. Fibers in piezoelectric composites could greatly benefit from coating technology, enhancing their electro-mechanical characteristics. Because devices made of piezoelectric composite materials are often subjected to dynamic loads, it is very interesting to study how waves move through these materials and how covered fiber-reinforced composites made of different piezoelectric materials cause waves to spread out^6,7^.

Piezoelectric materials are used extensively in fields as diverse as medicine and space travel. They are also used in intelligent structure systems, MEMS, accelerometers, acoustic and pressure sensing, precision controller design, sensors for monitoring, ultrasonic transducers, piezoelectric composite systems, sound systems, and headphones. Since the 1800s, scientists have tried to figure out what causes the electric and magnetic responses of a material to be linked to its thermomechanical response. Piezoelectric materials were initially used in hydrophones in the mid-twentieth century. The idea of composite materials that combine electrical and magnetic properties has emerged in the last two decades. These composites may display field coupling not seen in the individual components. The application of so-called composite and smart materials could be helpful for a wide range of developing vehicles, including optical and ultrasound sensors, gyroscopes, electric motors, and transducers, among many others. There are many uses for magneto-elastic materials. These materials have found utility in cutting-edge fields like lasers, supersonic gadgets, microwaves, and infrared purposes due to their ability to transform energy types^8^. In addition, ferroelectric composites are inherently anisotropic and exhibit correlated behaviors between mechanical, electrical, and electromagnetic interactions. Several researchers have taken advantage of the generalized theories of thermoelasticity to investigate some issues related to the motion of waves through magneto-thermoelastic and thermoelastic materials. Using the dual-phase-lag model, Ahmed et al.^9^ looked at how rotation affected the transmission of a plane wave through a half-space of a piezo-thermoelastic medium. Li and He^10^ considered the case of a comprehensive piezoelectric thermoplastic, which involves a flexible piezoelectric rod with a finite length, temperature-dependent properties, and exposure to a moving heat source. Generalized thermoelasticity, which incorporates the theory of non-local elasticity and the law of fractional thermal conductivity, was used to study this problem.

The idea of FGM materials was first presented in 1984 in Japan as part of the spaceplane project as a thermal barrier material. In the past few years, there has been more interest in studying solid mechanics problems where the flexible parameters are not constants but depend on where they are. Variation of elastic modulus due to inhomogeneity is a more realistic scenario and thus an inspiration for this study. The qualities of an FGM vary with its volume because its composition and structure change with time. Instead of using traditional homogeneous materials, FGMs often prove to be superior in a variety of settings. Components of aircraft engineering, turbines, and spacecraft are vulnerable to catastrophic failure when subjected to thermal shocks and very high temperatures, which generate severe thermoelastic strains^11^. Microscopically heterogeneous functionally graded materials (FGMs) are composite materials whose overall properties keep changing in one (or more) directions to reduce the effects of single stresses, lower residual stresses, and improve bonding strength. Because of this, FGMs are being used in a wide range of fields, including aerospace, automotive, marine, and biology^12^. Still, the design of FGMs depends a lot on their effective characteristics and, more importantly, how these characteristics relate to the microstructure to get the needed performance. Because of this, it is important to be able to predict the mechanical, thermal, and other qualities based on the material's microstructure and how it spreads^13^.

The effectiveness of FGMs depends not only on the types and amounts of materials used but also on how well the designer knows how to use those materials in the best way possible. One of the most important steps in designing FG systems is to accurately simulate how they will respond to the complex thermomechanical loads of those materials in the best way possible. The thermomechanical response to FGM material has been the focus of many analytical studies in recent years and has typically included fairly simplified geometries, material properties, and boundary conditions.

Peng et al.^14^ investigated an FGM microbeam's non-local extended thermal flexibility and thermally induced transient behavior. The material is heated by a ramp-style heating load at the left end of the microbeam. To assess thermoelastic phenomena, including temperature distribution, tension, and thermal stress, Go^15^ considered FG rotating circular disks. Generalized thermoelastic functionals with gradations were studied by Abo-Dahab et al.^16^ using a non-gaussian laser beam shaped like a narrow strip. Abouelregal and Dargail^17^ brought attention to the fact that a novel mathematical formulation for FG thermally induced nanobeams (FGNB) with a customizable kernel function and delay period has been introduced. Considering the thermal sensitivity of materials, Yevtushenko et al.^18^ devised a version of the frictional thermal treatment technique that occurs throughout single braking to calculate the temperature change of the FG friction parts. Utilizing the more comprehensive theory of thermoelasticity, Abouelregal et al.^19,20^ investigated the thermoelastic waves of an FG thermo-piezoelectric fixed rod. The thermo-piezoelectric rod is grounded at both ends and heated by a variable axial element.

When the heat transfer equation is studied for thermoelastic materials, it can be improved by considering their physical properties. This is because it is used in modern engineering applications. Many researchers have used fractional calculus to modify the heat transfer equation within the concept of extended thermoelasticity. When dealing with extended thermoelasticity with thermal relaxation periods, memory-dependent (MDD) calculus may be preferred over fractional calculus^21^. This is due to the fact that MDD differentiates the present rate of change, which is dependent on the preceding state. MDD can also be expressed as an integral function with a kernel function and derivatives in common. Consequently, when the time delay in the heat transfer model and the kernel function of the integral form is elastic, a model with MDD is utilized for various engineering applications. The MDD concept is preferable to fractional derivatives in this instance^22^.

Wang and Li^22^ presented an article on the idea of MDD by applying the fractional order derivative to different realistic theories. As an extension of the effort previously produced by Wang and Li^22^, Yu et al.^23^ presented a paper discussing a novel modified thermoelasticity framework using the MDD. Peng et al.^24^ developed an innovative microscale thermo-viscoelastic nonlocal model that accounts for the memory-dependent and size-dependent effects on polymer microbeam responses. This model takes into account the impacts of the kernel function, time delay, viscous damping parameter, and fractional order coefficient. To describe damping in many oscillatory systems of complicated dissipation appliances in which memory influences could not be ignored, Al-Jamel et al.^25^ developed a memory-dependent derivative with respect to displacement. Using a two-temperature thermoelastic model, Kaur et al.^26^ investigated the effect of memory-dependent derivatives (MDD) on a two-dimensional isotropic thermoelastic material in the existence of a magnetic field. Kaur and Singh^27^ presented a modified couple stress theory for a fiber-reinforced magneto-thermoelastic material with ramp-type distributed heat, Hall current, and hyperbolic two-temperature. A mathematical representation of the problem is constructed utilizing Fourier's law of heat transfer with fractional order and three-phase delay derivatives. Based on a memory-dependent concept, Kaur and Singh^28^ analyzed the thermoelastic vibrations in 2D functionally graded nanobeams (FGN). The nanobeam has a constant temperature in the middle and is sustained at both ends. The FGN's composite structure varies continuously in thickness, from ceramic at the base to metal at the surface, making it anything but homogeneous. Kaur and Singh^29^ studied the thermoelastic changes in a thick circular plate with transverse isotropy under ring loading. Two-temperature models based on modifying the Green Naghdi (GN) heat conduction equation accounting for memory-dependent derivatives (MDD) have been employed to study the phenomenon. Kaur et al.^30^ studied the properties of piezo-thermoelastic nanobeams and other one-dimensional materials with piezoelectric properties. The theoretical framework was developed using a combination of nonlocal Eringen's concept and an extended piezo-thermo-elastic framework at two temperatures.

During the building and reaction processes, intelligent structures are often exposed to both constant and intermittent sources of heat. Therefore, it is crucial for intelligent structural design methods to have an in-depth understanding of the effects, thermal response, and piezoelectric response of functionally scaled structures. To the authors' knowledge, there is no prior literature that addresses rotating the thermo-piezoelectricity of materials with functional grades under generalized thermoelasticity theory. The topic of this study is a one-dimensional perturbation in an isotropic finite bar within the framework of a comprehensive thermoelectric model with one relaxation period in the presence of variable, moving, and dissipating heat sources. The functionally graded material's (FGM) characteristics are expected to vary dramatically with distance.

As an improvement of the traditional coupled thermoelasticity theory, this proposed work will present general equations describing functionally graded thermo-piezoelectric materials based on the Lord and Shulman model incorporating memory-dependent derivatives. The mathematical framework was employed to study the propagation of heat piezoelectric waves through a heterogeneous rotating rod composed of an inner and outer piezoelectric layer connected by linear elastic materials. The relevant physical quantities, such as deformations, thermoelectric stress, temperature change, and electric potential, can be solved using the successive separation method and the Laplace transform. The different domains can be changed into the time domain using an appropriate approximation algorithm. Also shown are the stress and electric displacement obtained numerically for various nonhomogeneity indices and thermoelastic models. Finally, the findings are contrasted with those already published in the scientific literature. In particular, it is seen that by setting the non-homogeneity index to zero, the outcomes of the corresponding homogeneous situation may be reconstructed from the obtained results. The current findings may have applications in developing various pyro/piezoelectric devices, including sensors and gyroscopes with piezoelectric components.

## Governing system of equations

It is possible to formulate equations that govern thermo-piezoelectricity theory for piezoelectric materials under a thermal field using the following system equations^31,32^:

The constitutive equations and strains:

1 $${T}_{ij}={C}_{ijkl}{S}_{kl}-{e}_{ijk}{E}_{k}-{\beta }_{ij}\theta$$

2 $$2{S}_{ij}={u}_{i,j}+{u}_{j,i}$$

The entropy equation

3 $$\rho \eta =\frac{\rho {C}_{E}}{{T}_{0}}\theta +{\beta }_{ij}{S}_{ij}+{p}_{i}{E}_{i}$$

The energy equation

4 $$\rho {T}_{0}\frac{\partial \eta }{\partial t}-\rho Q+{h}_{i,i}=0$$

A concept is provided to detect the thermomechanical perturbation caused by a change in the electric potential and the modulation of the structure interaction by applying electric fields to the thermo-piezoelectric elastic field, and the result will be piezo-thermoelastic components. Mindlin^31^ is credited with introducing the idea of the thermo-piezoelectric effect and deriving the equations of motion for the thermo-piezoelectric plate. Nowacki^33^ has researched thermo-piezoelectric materials and the principles governing their physical characteristics. Relationships describing electrical displacement are provided by

5 $${D}_{i}={e}_{ijk}{S}_{kl}+{\in }_{ij}{E}_{j}+{p}_{i}\theta$$

The effect of heat flow rate was taken into account in constructing Fourier's law in the Lord and Shulman model by presenting a new physical coefficient called the relaxation time ${\tau }_{0}$. According to a revised version of Lord and Shulman's Fourier's Law, heat conduction can be expressed as

6 $${h}_{i}+{\tau }_{0}\frac{\partial {h}_{i}}{\partial t}=-{K}_{ij}{\theta }_{,j},\text{\hspace{0.17em}\hspace{0.17em}\hspace{0.17em}\hspace{0.17em}}i,j,k=1,2,3$$

The finite speeds at which the displacement and temperature waves travel can be calculated using these equations.

To illustrate the part memory plays in thermoelasticity theory, Yu et al.^23^ incorporated the concept of memory-dependent derivatives (MDD) into Lord Shulman's modified thermoelastic model with heat flow rate. In this proposed model, Fourier's law takes the following form:

7 $$\left(1+{\tau }_{0}{D}_{\omega }\right){h}_{i}=-{K}_{ij}{\theta }_{,j},$$

where ${D}_{\omega }$ represents the MDD of the first order, which is defined as follows^22^:

8 $${D}_{\omega }^{(1)}{h}_{i}\left(x,t\right)=\frac{1}{\omega }{\int }_{t-\omega }^{t}k\left(t-\xi \right){h}_{i}^{{{\prime}}}(x,\xi )d\xi$$

Also, $\omega >0$ symbolizes the time delay, and $k\left(t-\xi \right)$ is a freely selectable kernel function with $0\le k\left(t-\xi \right)\le 1$. For piezo-thermoelastic solids, we can establish the modifiedMDD heat transport equation with a single-phase lag by using Eqs. (3), (4), and (7) as

9 $${\left({K}_{ij}{\theta }_{,j}\right)}_{,i}=\left(1+{\tau }_{0}{D}_{\omega }\right)\left(\rho {C}_{E}\frac{\partial \theta }{\partial t}+{\beta }_{ij}\text{\hspace{0.17em}}{T}_{0}\frac{\partial {u}_{m,m}}{\partial t}-{T}_{0}{p}_{k}{\dot{E}}_{k}-\rho Q\right)$$

The piezoelectric problem is controlled when there is no external force on the body or free charges by using the following equations^34^:

10 $${D}_{i,i}=0, {E}_{i}=-{\varphi }_{,i}$$

It is possible to express the equations of motion as^35^

11 $${C}_{ijkl}{u}_{k,li}+{e}_{kij}{\varphi }_{,ki}-{\beta }_{ij}{\theta }_{,i}\text{=}\rho \text{\hspace{0.17em}}{\ddot{u}}_{i}$$

Also, introducing Gauss's divergence Eq. (5) into Eq. (10) gives

12 $${e}_{kij}{u}_{i,jk}-{\in }_{ij}{\varphi }_{,ij}+{p}_{i}{\theta }_{,i}=0$$

Assuming zero piezoelectric influences (${e}_{ijk}$, ${\in }_{ij}{p}_{i}\to 0$), the fundamental system field equations simplify to modified thermoelasticity with a relaxation time. Moreover, the coupled thermo-piezoelectricity framework equations are obtained by ignoring the thermal relaxation time (${\tau }_{0}\to 0$). It is also possible to obtain the conventional Fourier heat transport if the parameters of piezoelectricity and relaxation time are all zero (${e}_{ijk},{\in }_{ij},{p}_{i},{\beta }_{ij}\to 0$ and ${\tau }_{0}\to 0$).

## Problem formulation

**Figure 1:**
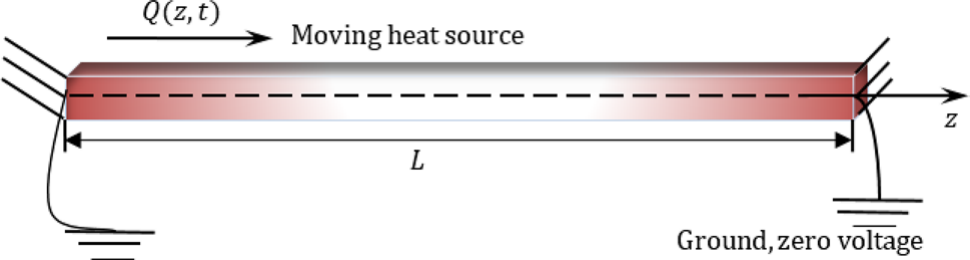
A pictorial representation of a piezoelectrically rotating flexible rod.

Here, we focus on the case of a finite rod of thermoelastic material with a functional gradation occupying the region $0\le z\le L$. Considering that the origin of the coordinates at the left edge of the piezoelectric elastic rod, as displayed in Fig. 1, was the alignment of the rod along the $z$-axis. In addition to being permanently attached, there is insulation on both ends to prevent heat loss. Inthe end,$z=0$, we assume a flat distribution of a heat source $Q(z,t)$ moving steadily towards the right. We will suppose that the rod was initially motionless and at a temperature of ${T}_{0}$. Torsional deformation occurs when the structure is subjected to a torque moment about its axis. In the current problem, it has been taken into account that the studied rod is long and slender and is not subject to torque moments around its long axis. Hence, the torsion deformation was neglected. For long beams, the shear deformations of the rods are negligible. It is assumed that the physical characteristics of the rod vary exponentially across its axial length.

The material will be assumed to rotate at a constant angular velocity $\overrightarrow{\Omega }=\Omega \overrightarrow{\mathrm{n}}$, where $\overrightarrow{\mathrm{n}}$ represents the unit vector along the axis of rotation. The equation of motion has been expanded to incorporate two new terms as a consequence of the rotation process. The first term accounts for the gravitational acceleration ($\overrightarrow{\Omega }\times \left(\overrightarrow{\Omega }\times \overrightarrow{\mathrm{u}}\right)$) caused by time-varying motion alone. In contrast, the second term represents the Coriolis acceleration of $2\overrightarrow{\Omega }\times \overrightarrow{\dot{\mathrm{u}}}$, where $\overrightarrow{\mathrm{u}}$ is the displacement vector. The distortion is also assumed to be significantly small. Due to the nature of the problem, only the spatial variable $z$ and the time variable $t$ are necessary to describe the state of the rod during movement and rotation. Also, it will be assumed that the polarization of the piezoelectric rod works in a direction that is parallel to the direction of the rod.

In order to get a clearer assessment of the one-dimensional issue, we will suppose that the electric field ${E}_{z}$, displacement $w$, strain ${S}_{zz}$, thermal stress ${T}_{zz}$, heat flow $Q$, and electric displacement $D$ are all functions of $z$ and $t$ only. The thermo-piezoelectric governing equations for a one-dimensional situation can be expressed as

13 $${S}_{zz}\left(z,t\right)=\frac{\partial w\left(z,t\right)}{\partial z},\text{\hspace{0.17em}\hspace{0.17em}\hspace{0.17em}\hspace{0.17em}\hspace{0.17em}\hspace{0.17em}\hspace{0.17em}\hspace{0.17em}\hspace{0.17em}\hspace{0.17em}\hspace{0.17em}}{E}_{z}\left(z,t\right)=-\frac{\partial \varphi \left(z,t\right)}{\partial z}$$

14 $${T}_{zz}(z,t)=C\frac{\partial w}{\partial z}+e\frac{\partial \varphi }{\partial z}-\beta \theta$$

15 $$\frac{\partial {T}_{zz}}{\partial z}=\rho \left[\frac{{\partial }^{2}}{\partial {t}^{2}}-{\Omega }^{2}-2\Omega \frac{\partial }{\partial t}\right]w$$

16 $${D}_{z}(z,t)=e\frac{\partial w}{\partial z}-\in \frac{\partial \varphi }{\partial z}+p\theta$$

17 $$\frac{\partial }{\partial z}\left(K\frac{\partial \theta }{\partial z}\right)=\left(1+{\tau }_{0}{D}_{\omega }\right)\left(\rho {C}_{E}\frac{\partial \theta }{\partial t}+\beta \text{\hspace{0.17em}}{T}_{0}\frac{\partial w}{\partial t\partial z}+{T}_{0}p\frac{\partial \varphi }{\partial t\partial z}-\rho Q\right)$$

Equation (8) gives $\frac{\partial D}{\partial z}=0$, which implies that the electrical displacement is assumed to be

18 $$D(z,t)={D}_{0}={\text{constant}}.$$

Substitution of Eq. (18) into Eq. (16) results in

19 $$\frac{\partial \varphi }{\partial z}=\frac{e}{\in }\frac{\partial w}{\partial z}-\frac{{D}_{0}}{\in }+\frac{p}{\in }\theta$$

As a result of plugging Eq. (19) into Eqs. (14) and (17), we obtain:

20 $${T}_{zz}=\left(C+\frac{{e}^{2}}{\in }\right)\frac{\partial w}{\partial z}-\frac{e{D}_{0}}{\in }+\left(\frac{ep}{\in }-\beta \right)\theta$$

21 $$K\frac{{\partial }^{2}\theta }{\partial {z}^{2}}+\frac{\partial K}{\partial z}\frac{\partial \theta }{\partial z}=\left(1+{\tau }_{0}{D}_{\omega }\right)\left(\left(\rho {C}_{E}+\frac{{T}_{0}{p}^{2}}{\in }\right)\frac{\partial \theta }{\partial t}+{T}_{0}\left(\beta +\frac{{p}^{2}}{\in }\right)\text{\hspace{0.17em}}\frac{\partial w}{\partial t\partial z}-\rho Q\right)$$

Many publications discussing the mechanical properties of FGMs have been published in recent years. FGMs are a type of material whose characteristics change continually with location. Studies often focus on particle composites, where the dispersed phase's volume fraction fluctuates continuously with thickness. The following formula describes how matter's physical properties evolve exponentially in the $z$-axis direction^8^:

22 $$\Psi (z)={\Psi }_{0}{e}^{\xi z}$$

where $\Psi (z)$ represents the change inphysical properties, ${\Psi }_{0}$ is assumed to befixedandreflects the property that a substance has when $z$ is equal to zero (homogeneous material), and $\xi$ is an indicator of heterogeneity. When we plug the relationship (22) into the governing Eqs. (19)–(21), we get

23 $$\frac{\partial \varphi }{\partial z}=\frac{{e}_{0}}{{\in }_{0}}\frac{\partial w}{\partial z}+\frac{{p}_{0}}{{\in }_{0}}\theta -\frac{{D}_{0}}{{\in }_{0}}{e}^{-\xi z}$$

24 $${T}_{zz}={e}^{\xi z}\left[\left({C}_{0}+\frac{{e}_{0}^{2}}{{\in }_{0}}\right)\frac{\partial w}{\partial z}+\left(\frac{{e}_{0}{p}_{0}}{{\in }_{0}}-{\beta }_{0}\right)\theta \right]-\frac{{e}_{0}{D}_{0}}{{\in }_{0}}$$

25 $${K}_{0}\left(\frac{{\partial }^{2}\theta }{\partial {z}^{2}}+\xi \frac{\partial \theta }{\partial z}\right)=\left(1+{\tau }_{0}{D}_{\omega }\right)\left(\left({\rho }_{0}{C}_{E}+\frac{{T}_{0}{p}_{0}^{2}}{{\in }_{0}}\right)\frac{\partial \theta }{\partial t}+{T}_{0}\left({\beta }_{0}+\frac{{p}_{0}^{2}}{{\in }_{0}}\right)\text{\hspace{0.17em}}\frac{\partial w}{\partial t\partial z}-{\rho }_{0}Q\right)$$

When the body is examined for the phenomenon of heat transfer, moving heat sources are those physical situations in which thermal excitation regularly changes its location and intensity. Assume we have a stationary heat source with an intensity ${Q}_{0}$ that is turned on at time $t=0$ and moves continuously along the $z$-axis with a constant velocity^36^:

26 $$Q(z,t)={Q}_{0}\delta \left(z-\upsilon t\right)$$

where the function $\delta (.)$ denotes the Dirac delta.

For simplicity, the following dimensionless variable quantities have been provided:

27 $$\begin{array}{c}({z}^{{{\prime}}},{w}^{{{\prime}}})=\omega \eta (z,w),\text{\hspace{0.17em}}\text{\hspace{0.17em}}\text{\hspace{0.17em}}({t}^{{{\prime}}},{\tau }_{0}^{^{\prime}})={\omega }^{2}\eta (t,{\tau }_{0}),\text{\hspace{0.17em}}\text{\hspace{0.17em}}\text{\hspace{0.17em}}{\theta }^{{{\prime}}}=\frac{\theta }{{T}_{0}},\text{\hspace{0.17em}}\text{\hspace{0.17em}}\text{\hspace{0.17em}}{T}_{zz}^{^{\prime}}=\frac{{T}_{zz}}{{C}_{0}},\\ {D}_{z}^{^{\prime}}=\frac{{D}_{z}}{{C}_{0}},{\varphi }^{{{\prime}}}=\frac{\omega \eta {\in }_{0}}{{e}_{0}}\varphi ,\text{\hspace{0.17em}}\text{\hspace{0.17em}}\text{\hspace{0.17em}}\text{\hspace{0.17em}}{Q}^{{{\prime}}}=\frac{Q}{{\omega }^{2}{\eta }^{2}{K}_{0}{T}_{0}},\text{\hspace{0.17em}}\text{\hspace{0.17em}}\text{\hspace{0.17em}}\text{\hspace{0.17em}}\text{\hspace{0.17em}}{\omega }^{2}=\frac{{C}_{0}}{{\rho }_{0}},\text{\hspace{0.17em}}\text{\hspace{0.17em}}\text{\hspace{0.17em}}\text{\hspace{0.17em}}\text{\hspace{0.17em}}\eta =\frac{{\rho }_{0}{C}_{E}}{{K}_{0}},\text{\hspace{0.17em}}l=Lv\eta .\end{array}$$

By removing the primes, we can reformulate Eqs. (23), (24), and (25) as

28 $$\frac{\partial \varphi }{\partial z}=\frac{\partial w}{\partial z}+{H}_{12}\theta -{Hh}_{13}{e}^{-Nz}$$

29 $${T}_{zz}={e}^{Nz}\left[{H}_{21}\frac{\partial w}{\partial z}-{H}_{22}\theta \right]-{H}_{23}$$

30 $$\left(\frac{{\partial }^{2}\theta }{\partial {z}^{2}}+N\frac{\partial \theta }{\partial z}\right)=\left(1+{\tau }_{0}{D}_{\omega }\right)\left({H}_{31}\frac{\partial \theta }{\partial t}+{H}_{32}\text{\hspace{0.17em}}\frac{\partial w}{\partial t\partial z}-Q\right)$$

where

31 $$\begin{array}{c}{H}_{12}=\frac{{T}_{0}{p}_{0}}{{e}_{0}},\text{\hspace{0.17em}}\text{\hspace{0.17em}}\text{\hspace{0.17em}}\text{\hspace{0.17em}}{H}_{13}=\frac{{C}_{0}{D}_{0}}{{e}_{0}},\text{\hspace{0.17em}}\text{\hspace{0.17em}}N=\frac{\xi }{\omega \eta },\text{\hspace{0.17em}}\text{\hspace{0.17em}}{H}_{21}=\frac{{C}_{0}{\in }_{0}+{e}_{0}^{2}}{{C}_{0}{\in }_{0}},\text{\hspace{0.17em}}{H}_{23}=\frac{{e}_{0}{D}_{0}}{{\in }_{0}},\text{\hspace{0.17em}}\\ \text{\hspace{0.17em}}{H}_{22}=\frac{{T}_{0}\left({\in }_{0}{\beta }_{0}-{e}_{0}{p}_{0}\right)}{{C}_{0}{\in }_{0}},\text{\hspace{0.17em}}\text{\hspace{0.17em}}\text{\hspace{0.17em}}\text{\hspace{0.17em}}{H}_{31}=\frac{{K}_{0}\eta {\in }_{0}+{T}_{0}{p}_{0}^{2}}{{K}_{0}\eta {\in }_{0}},\text{\hspace{0.17em}}\text{\hspace{0.17em}}{H}_{32}=\frac{{\beta }_{0}}{{K}_{0}\eta }+\frac{{p}_{0}^{2}}{{K}_{0}\eta {\in }_{0}}.\end{array}$$

When Eq. (29) is put into Eq. (15), the piezo-thermoelasticity motion equation is

32 $${H}_{21}\left(\frac{{\partial }^{2}w}{\partial {z}^{2}}+N\frac{\partial w}{\partial z}\right)-{H}_{22}\left(\frac{\partial \theta }{\partial z}+N\theta \right)={\rho }_{0}{\omega }^{3}\eta \left[\frac{{\partial }^{2}}{\partial {t}^{2}}-{\Omega }^{2}-2\Omega \frac{\partial }{\partial t}\right]w$$

## Solution technique

It is assumed that the following can be written as the problem's initial conditions:

33 $$w(z,0)=0=\dot{w}(z,0),\text{\hspace{0.33em}\hspace{0.33em}}\theta (z,0)=0=\dot{\theta }(z,0).$$

Using the relationship in Eq. (33) and the Laplace integral transform in Eqs. (28)–(30), we get

34 $$\frac{d\overline{\varphi }}{dz }=\frac{d\overline{w}}{dz }+{H}_{12}\overline{\theta }-{H}_{13}{e}^{-Nz}/s$$

35 $${\tilde{\sigma }}_{zz}={e}^{Nz}\left[{H}_{21}\frac{d\overline{w}}{dz }-{H}_{22}\overline{\theta }\right]-{H}_{23}/s$$

36 $$\frac{{d}^{2}\overline{\theta }}{d{z}^{2}}+N\frac{d\overline{\theta }}{dz}=s\left(1+\frac{{\tau }_{0}}{\omega }\overline{G }\left(s,\omega \right)\right)\left({H}_{31}\overline{\theta }+{H}_{32}\text{\hspace{0.17em}}\frac{dw}{dz}-\frac{{Q}_{0}}{\upsilon }{e}^{-(s/\upsilon )z}\right)$$

37 $${H}_{21}\left(\frac{{d}^{2}\overline{w}}{d{z }^{2}}+N\frac{d\overline{w}}{dz }\right)-{H}_{22}\left(\frac{d\overline{\theta }}{dz}+N\overline{\theta }\right)={\rho }_{0}{\omega }^{3}\eta \left({s}^{2}-{\Omega }^{2}-2{\Omega {\mathrm{s}}}\right)\overline{w }$$

The kernel function $k\left(\mathrm{t}-\upxi \right)$ can be selected randomly as^35^

38 $$k\left(t-\xi \right)=1-\frac{2b\left(t-\xi \right)}{\omega }+\frac{{a}^{2}}{{\omega }^{2}}{\left(t-\xi \right)}^{2}$$

The parameters $a$ and $b$ are constants that can be selected according to the shape of the kernel. Any first-order MDD function $g\left(t\right)$ has a Laplace transform, which is defined as:

39 $$\begin{array}{c}L\left[\omega {D}_{\omega }g\left(t\right)\right]=L\left[{\int }_{t-\omega }^{t}k\left(t-\xi \right){g}^{{{\prime}}}\left(\xi \right)d\xi \right]=L\left[g(t)\right]\overline{G }\left(s,\omega \right);\\ \overline{G }\left(s,\omega \right)=({1-e}^{-s\omega }) \left[1-\frac{2b}{\omega s}+\frac{2{a}^{2}}{{\omega }^{2}{s}^{2}}\right]-\left[{a}^{2}-2{b}^{2}+\frac{2{a}^{2}}{\omega s}\right]{e}^{-s\omega }\end{array}$$

If the kernel $k\left(t-\xi \right)=1$ then we have

40 $$\overline{G }\left(s,\omega \right)=\left({1-e}^{-s\omega }\right)$$

It is possible to rewrite Eqs. (36) and (37) as:

41 $$\left(\frac{{d}^{2}}{d{z}^{2}}+N\frac{d}{dz}-{H}_{41}\right)\text{\hspace{0.17em}}\overline{\theta }={H}_{42}\frac{dw}{dz}-{H}_{43}{e}^{-(s/\upsilon )z}$$

42 $${H}_{22}\left(\frac{d}{dz}+N\right)\text{\hspace{0.17em}}\overline{\theta }={H}_{21}\left(\frac{{d}^{2}}{d{z}^{2}}+N\frac{d}{dz}-{H}_{44}\right)\overline{w }$$

where

43 $$\begin{array}{c}{H}_{41}=s\left(1+\frac{{\tau }_{0}}{\omega }\overline{G }\left(s,\omega \right)\right){H}_{31},\text{\hspace{0.17em}}\text{\hspace{0.17em}}{H}_{42}=s\left(1+{\tau }_{0}s\right){H}_{32},{H}_{43}=s\left(1+\frac{{\tau }_{0}}{\omega }\overline{G }\left(s,\omega \right)\right){Q}_{0}/\upsilon ,\\ \text{\hspace{0.17em}}\text{\hspace{0.17em}}{H}_{44}={\rho }_{0}{\omega }^{3}\eta \left({s}^{2}-{\Omega }^{2}-2{\Omega {\mathrm{s}}}\right)/{H}_{21}.\end{array}$$

The first and second-order derivatives of Eq. (41) are substituted into Eq. (42) to yield the displacement ODE shown below:

44 $$\left(\frac{{d}^{4}}{d{z}^{4}}+{\psi }_{3}\frac{{d}^{3}}{d{z}^{3}}-{\psi }_{2}\frac{{d}^{2}}{d{z}^{2}}-{\psi }_{1}\frac{d}{dz}+{\psi }_{0}\right)\text{\hspace{0.17em}}\overline{w }={m}_{0}{e}^{-(s/\upsilon )z}$$

where

45 $$\begin{array}{c}{\psi }_{3}=2N,\text{\hspace{0.17em}}\text{\hspace{0.17em}}\text{\hspace{0.17em}}\text{\hspace{0.17em}}\text{\hspace{0.17em}}\text{\hspace{0.17em}}\text{\hspace{0.17em}}{\psi }_{2}={H}_{41}+{N}^{2}+{H}_{44}+{H}_{42}{H}_{22}/{H}_{21},\text{\hspace{0.17em}}\text{\hspace{0.17em}}{\psi }_{0}={H}_{44}{H}_{41}\\ {\psi }_{1}=N{H}_{41}+N{H}_{44}+N{H}_{42}{H}_{22}/{H}_{21},{m}_{0}={H}_{43}{H}_{22}\left(s/v-N\right)/{H}_{21}\text{\hspace{0.17em}}.\end{array}$$

We can write the characteristic equation of the ODE equation (44) as

46 $${k}^{4}+{\psi }_{3}{k}^{3}-{\psi }_{2}{k}^{2}-{\psi }_{1}k+{\psi }_{0}=0$$

where the roots ${k}_{i}$, ${\mathrm{i}}=1,2,3,4$ can be found as follows:

47 $$\begin{array}{c}{k}_{1}=-\frac{{\psi }_{3}}{4}-\frac{{y}_{6}}{2}-\frac{{y}_{8}}{2},\text{\hspace{0.17em}}\text{\hspace{0.17em}}\text{\hspace{0.17em}}\text{\hspace{0.17em}}\text{\hspace{0.17em}}{k}_{2}=-\frac{{\psi }_{3}}{4}-\frac{{y}_{6}}{2}+\frac{{y}_{8}}{2},\text{\hspace{0.17em}}\text{\hspace{0.17em}}\text{\hspace{0.17em}}\\ {k}_{3}=-\frac{{\psi }_{3}}{4}+\frac{{y}_{6}}{2}-\frac{{y}_{8}}{2},\text{\hspace{0.17em}}\text{\hspace{0.17em}}\text{\hspace{0.17em}}\text{\hspace{0.17em}}\text{\hspace{0.17em}}\text{\hspace{0.17em}}{k}_{4}=-\frac{{\psi }_{3}}{4}+\frac{{y}_{6}}{2}+\frac{{y}_{8}}{2},\end{array}$$

with

48 $$\begin{array}{c}{y}_{0}=12{\psi }_{0}+{\psi }_{2}^{2}+3{\psi }_{1}{\psi }_{3},\text{\hspace{0.17em}}{y}_{1}=27{\psi }_{1}^{2}+72{\psi }_{0}{\psi }_{2}-2{\psi }_{2}^{3}-9{\psi }_{1}{\psi }_{2}{\psi }_{3}+27{\psi }_{0}{\psi }_{3}^{2},\\ {y}_{2}=\frac{2{\psi }_{2}}{3}+\frac{{\psi }_{3}^{2}}{4}, {y}_{3}=8{\psi }_{1}-4{\psi }_{2}{\psi }_{3}-{\psi }_{3}^{3},\text{\hspace{0.17em}}\text{\hspace{0.17em}}\text{\hspace{0.17em}}{y}_{4}=\sqrt[3]{{y}_{1}+\sqrt{-4{y}_{0}^{3}+{y}_{1}^{2}}}/3\sqrt[3]{2},\\ {{y}_{8}=\sqrt{{y}_{7}/4\sqrt{{y}_{6}}}, y}_{5}=\left(\sqrt[3]{2}{y}_{0}/3\right)/\sqrt[3]{{y}_{1}+\sqrt{-4{y}_{0}^{3}+{y}_{1}^{2}}},\text{\hspace{0.17em}}\text{\hspace{0.17em}}\\ {y}_{7}={y}_{2}-\left({y}_{5}+{y}_{4}+{y}_{3}\right),\text{\hspace{0.17em}}\text{\hspace{0.17em}}{y}_{6}=\sqrt{{y}_{2}+{y}_{5}+{y}_{4}}.\end{array}$$

The general solution to Eq. (44) describing an inhomogeneous system can be stated as

49 $$\overline{w} = \mathop \sum \limits_{i = 1}^{4} A_{i} e^{{k_{i} z}} + A_{5} e^{{ - \left( {s/\upsilon } \right)z}} .$$

The coefficients $A_{i}$, ${\text{i}} = 1,$ 2, 3, 4 denote the integral coefficients. In addition to that, the coefficient $A_{5}$ takes the following form

50 $$A_{5} = \frac{{\upsilon^{4} m_{0} }}{{s^{4} - a_{3} \upsilon s^{3} - a_{2} \upsilon^{2} s^{2} + \upsilon^{3} sa_{1} + \upsilon^{4} a_{0} }},$$

Similarly, by removing $\overline{w}$ from Eqs. (41) and (42), we obtain

51 $$\left( {\frac{{d^{4} }}{{dz^{4} }} + \psi_{3} \frac{{d^{3} }}{{dz^{3} }} - \psi_{2} \frac{{d^{2} }}{{dz^{2} }} - \psi_{1} \frac{d}{dz} + \psi_{0} } \right)\,\overline{\theta } = - m_{1} e^{{ - \left( {s/\upsilon } \right)z}} ,$$

where

52 $$m_{1} = H_{43} \left( {\left( {s/\upsilon } \right)^{2} - sN/\upsilon - H_{44} } \right)$$

In this case, we can express the temperature solution as:

53 $$\overline{\theta } = \mathop \sum \limits_{i = 1}^{4} B_{i} e^{{k_{i} z}} + B_{5} e^{{ - \left( {s/\upsilon } \right)z}} .$$

Using Eqs. (49) and (53) as replacements in Eq. (41), we can obtain:

54 $$\begin{aligned} B_{i} & = \frac{{H_{21} \left( {k_{i}^{2} + Nk_{i} - H_{44} } \right)}}{{H_{22} \left( {k_{i} + N} \right)}}A_{i} = \omega_{i} A_{i} , \quad i = 1,\;2,\;3,\;4 \\ B_{5} & = \frac{{H_{21} \left( {s^{2} /\upsilon^{2} - Ns/\upsilon - H_{44} } \right)}}{{H_{22} \left( { - s/\upsilon + N} \right)}}A_{5} = \omega_{5} A_{5} . \\ \end{aligned}$$

Consequently, the final solution for $\overline{\theta }$ may be expressed as

55 $$\overline{\theta } = \mathop \sum \limits_{i = 1}^{4} \omega_{i} A_{i} e^{{k_{i} z}} + \omega_{5} A_{5} e^{{ - \left( {s/\upsilon } \right)z}} .$$

Substituting (49) and (55) into Eq. (34), we then get

56 $$\frac{{d\overline{\varphi }}}{dz} = \mathop \sum \limits_{i = 1}^{4} F_{i} \,A_{i} e^{{k_{i} z}} + F_{5} \,e^{{ - \left( {s/\upsilon } \right)z}} - \left( {H_{13} /s} \right)e^{ - Nz} ,$$

where

57 $$F_{i} = k_{i} + H_{12} \omega_{i} ,\quad i = 1,2,3,4,\quad F_{5} = \left( { - s/\upsilon + H_{12} \omega_{5} } \right)A_{5} .$$

As a result, the Laplace transform domain solution for electric potential can be found as

58 $$\overline{\varphi } = \mathop \sum \limits_{i = 1}^{4} F_{i} \,A_{i} \,e^{{k_{i} z}} /k_{i} - \left( {\upsilon F_{5} /s} \right)\,e^{{ - \left( {s/\upsilon } \right)z}} + \left( {H_{13} \,/Ns} \right)e^{ - Nz} + A_{0}$$

The solutions for electric displacement $\overline{E}_{z}$ and normalized stress $\overline{T}_{zz}$ can be presented as:

59 $$\overline{T}_{zz} = e^{Nz} \left[ {\mathop \sum \limits_{i = 1}^{4} \left( {H_{21} k_{i} - H_{22} \omega_{i} } \right)A_{i} e^{{k_{i} z}} - \left( {H_{21} s/\upsilon + H_{22} \omega_{5} } \right)A_{5} e^{{ - \left( {s/\upsilon } \right)z}} } \right] - H_{23} /s,$$

60 $$\overline{E}_{z} = - \mathop \sum \limits_{i = 1}^{4} F_{i} \,A_{i} e^{{k_{i} z}} - F_{5} e^{{ - \left( {s/\upsilon } \right)z}} + \left( {H_{13} /s} \right)e^{ - Nz} ,$$

We will continue to assume that the rod is thermally insulated on both sides ($z = 0$ and $z = L$) and restricted (fixed)at zero voltage when $z = 0$. Therefore, the following criteria at the edges will be taken into account^36,37^:

61 $$\begin{aligned} & \varphi \left( {0,t} \right) = 0 \\ & w\left( {0,t} \right) = 0 = w\left( {L,t} \right) \\ & \frac{{\partial \theta \left( {0,t} \right)}}{\partial z} = 0 = \frac{{\partial \theta \left( {L,t} \right)}}{\partial z} \\ \end{aligned}$$

When applied to the boundary conditions, the Laplace transform (61) yields a set of equations in the unknown parameters $A_{i}$ where $i = 0,\;1,\;2,\;3,\;4$ as

62 $$A_{1} + A_{2} + A_{3} + A_{4} = - A_{5}$$

63 $$A_{1} e^{{k_{1} L}} + A_{2} e^{{k_{2} L}} + A_{3} e^{{k_{3} L}} + A_{4} e^{{k_{4} L}} = - A_{5} e^{{ - \left( {s/\upsilon } \right)L}}$$

64 $$A_{1} \omega_{1} + A_{2} \omega_{2} + A_{3} \omega_{3} + A_{4} \omega_{4} = - B_{5}$$

65 $$A_{1} \omega_{1} e^{{k_{1} L}} + A_{2} \omega_{2} e^{{k_{2} L}} + A_{3} \omega_{3} e^{{k_{3} L}} + A_{4} \omega_{4} e^{{k_{4} L}} = - B_{5} e^{{ - \left( {s/\upsilon } \right)L}}$$

66 $$\frac{{F_{1} }}{{k_{1} }}A_{1} + \frac{{F_{2} }}{{k_{2} }}A_{2} + \frac{{F_{3} }}{{k_{3} }}A_{3} + \frac{{F_{4} }}{{k_{4} }}A_{4} - \left( {\frac{{\upsilon F_{5} }}{s}\, - \frac{{H_{13} }}{Ns}} \right) + A_{0} = 0$$

By solving the above system equations, it is possible to set the values of the unknown parameters of integration ($A_{i}$, ${\text{i}} = 1,\;2,\;3,\;4$).

## Computational inversion of the transformed functions

The methods for calculating the inverse Laplace transform numerically are presented in this section. Over the past half-century, numerous strategies based on various perspectives have been offered. Numerical implementations of inverse Laplace transforms are not trivial to produce. This is what we call an "ill-conditioned" or "ill-posed" situation. Due to the lack of a universally applicable solution, we advocate employing a combination of approaches to any given inversion issue. We can have more confidence in a numerically computed inverse Laplace transform if two or more approaches obtain approximately the same result. Each of the several numerical inversion approaches shares the property that their performance improves in proportion to the smoothness of the original $g\left( t \right)$. We shall employ a numerically precise technique using the expansion of a Fourier series among these approaches^37^. We can use this method to return any $\overline{g}\left( s \right)$ function transformed using the following relation to the time domain $g\left( t \right)$:

67 $$g\left( t \right) = \frac{{e^{ct} }}{t}\left( {\frac{{\overline{g}\left( c \right)}}{2} + {\text{Re}}\mathop \sum \limits_{n = 1}^{{N_{0} }} \overline{g}\left( {c + in\pi /t} \right)\left( { - 1} \right)^{n} } \right)$$

$N_{0}$ is a finite integer, and the parameter $c$ has a value of $5 \le ct \le 10$^38^. The Laplace inversion formula is used for Eqs. (46), (52), and (54)–(56), yielding space–time domain solutions for the investigated field variables.

## Case studies

Using the technique explained in the previous section, the transmission of mechanical, thermal, and electrical vibrations in an FG piezoelectric rod will be studied. To prove the aforementioned analytical approach and verify the validity of the presented theoretical research results, the numerical state of a physical substance will be considered. An FG rod with cadmium selenide on the left end is considered for the numerical calculations. In our calculations, we have taken into account the following physical properties of the material^39^:

$$\begin{aligned} C_{0} & = 74.1 \times 10^{9} \;{\text{N/m}}^{2} ,\;\;\beta_{0} = 621 \times 10^{3} \;{\text{N/Km}}^{2} ,\;\;e_{0} = 0.347\;{\text{C/m}}^{2} ,\;\;T_{0} = 293\;{\text{K}}, \\ p_{0} & = - 2.94 \times 10^{ - 6} \;{\text{C/Km}}^{2} ,\;\;\rho_{0} = 7600\;{\text{kg}}\;{\text{m}}^{ - 3} ,\;\;Q_{0} = 10/\rho_{0} ,\;\;K_{0} = 12.9\;{\text{W/mK}}, \\ C_{E} & = 420\;{\text{J/kgK}},\; \in_{0} = 90.3 \times 10^{ - 12} \;{\text{C}}^{2} {\text{/Nm}},\;\;L = 1. \\ \end{aligned}$$

Some numerical results are given to study the theoretical results obtained in the previous sections. All data on the studied dimensionless domain variables were analyzed, and the figures were presented as a function of the axial distance. For several values of some factors, such as the speed of the applied heat supply,$\upsilon$, non-homogeneous parameter $N$, and the angular velocity of rotation ${\Omega }$, we examine the variations of the temperature change, $\theta$, electric potential, $\varphi$, normal thermal stress, $\sigma_{zz}$, and the displacement distribution $w$. When the moving heat source travels at high speeds, all points along the rod experience a nearly immediate evolution of the source's energy. This eliminates the temperature gradients throughout the rod, allowing it to be seen as a single entity for thermal purposes. The mathematical source term, $\delta \left( {z - \upsilon t} \right)$, changes to $\delta \left( t \right)$ at high velocities, $\upsilon$. The implication is that the original source loses its spatial dependence.

FG materials are heterogeneous materials specifically designed for high-temperature applications. This section highlights the importance of analytical studies focusing on the difficulties of transient thermoelasticity in such heterogeneous materials. In this particular instance, five distinct values of the non-homogeneous index $N$ are taken into consideration to discuss the impact that it has on the field variables that are being researched. For the FG material, we will use the values $N = 0.1$, $0.2$, and $0.3$,whereas the value $N = 0$ will represent the homogenous scenario with constant material attributes. The obtained results are presented graphically in graphs 2–5 in the case of $K_{3} = \left( {1 - \frac{t - \xi }{\omega }} \right)^{2}$ for the parameters $\omega = 0.01$, $\upsilon = 2$, ${\Omega } = 3$ and $\tau_{0} = 0.1$, which were kept constant. These figures significantly demonstrate that the gradient index $N$ significantly affects each investigated field.

**Figure 2:**
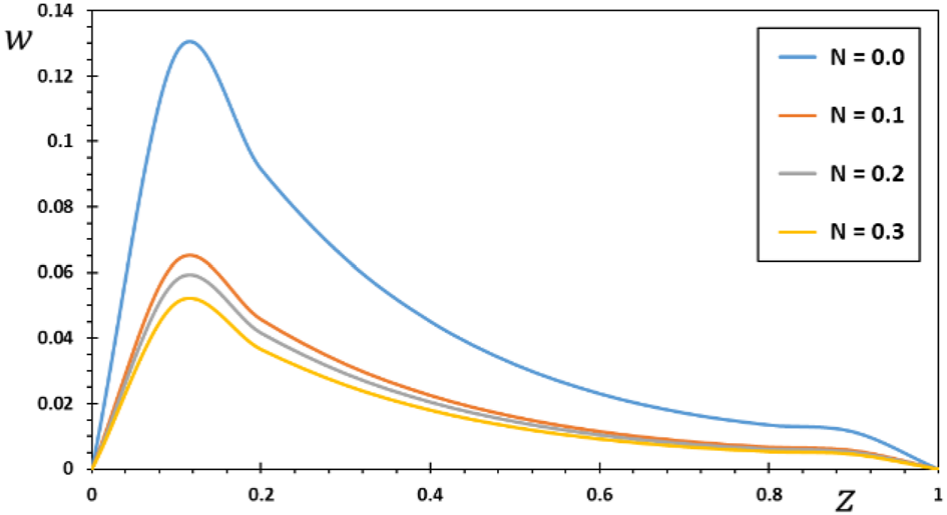
The displacement $w$ for various values of the gradient coefficient $N$.

Figure 2 illustrates how the material inhomogeneity ($N$) influences the displacement $w$. Since the two ends of the FG rod are fixed, the expansion deformation that occurs between the two ends is confined between them. This deformation results in compressive thermal stress in the rod. It is seen in Fig. 2 that the displacement at both ends of the FG rod meets the imposed boundary conditions where the displacement values $w = 0$ at $z = 0$ and $z = L$.

The medium is initially at an ambient temperature of $T_{0} = 293\;{\text{K}}$, after which it is exposed to a heat source moving at a constant speed. The source's energy production rate stays the same over the same amount of time. However, as the source velocity increases, the intensity of energy released per unit rod length diminishes. When the speed of the source increases, less energy is delivered to any one location. As a result, the interior of the penis cools less than the areas around it. As a result, the deformation increases at the beginning and then gradually decreases. Time slows or speeds up depending on the value of $z$, but as $\upsilon$ rises, energy from the source is released earlier in the timeline. As $\upsilon$ grows, this causes the rod temperature peak moves toward low $z$ values. Figure 2 shows that when the inhomogeneity parameter $N$ increases, the amount of displacement reduces. Figure 2 exhibits that as time passes, the temperature rises in the area of disturbance, which grows more extensively in the initial section of the piezoelectric rod. As time goes on, there may be an increasing amount of displacement because the rod has deformed due to thermal expansion as a result of the external source of heat. The thermally generated displacement and stress are also localized inside a restricted region due to the finite heat wave propagation, which limits the size of the region disturbed by heat at any given instant. This result aligns with the findings presented in^40–42^.

**Figure 3:**
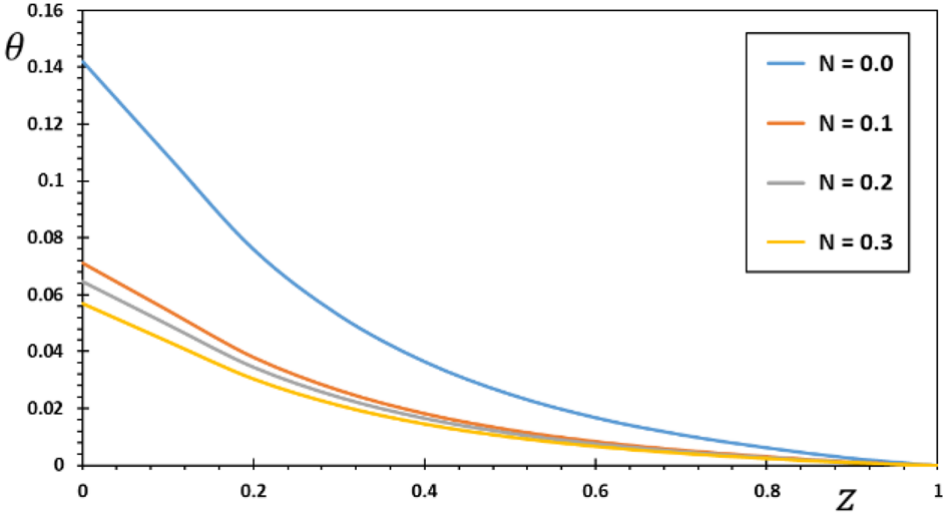
The temperature $\theta$ for different values of the gradient coefficient $N$.

Figure 3 shows the non-dimensional changes in temperature $\theta$ that occur in a piezoelectric rod for various options for the inhomogeneity coefficient $N$. We can see from Fig. 3 that the temperature $\theta$ decreases as both the amount of time elapsed and the distance traveled increase. It was also noted that the temperature $\theta$ reaches its maximum at the first end, where the source of the heat is, before decreasing towards the other end of the rod, indicating that the temperature is spreading at a limited speed in the rod. This completely differs from the conventional heat transfer theory, which predicts an unlimited speed. We also see that the amplitude of temperature $\theta$ is greatest in the homogeneous area where $N$ equals zero and decreases when the parameter $N$ is increased. As the source rate rises, the intensity of energy released per unit rod length diminishes. As a result, the amount of energy reaching any spot in the thermally turbulent region decreases proportionally as the source velocity rises. As a result, the temperature gradient inside the rod decreases locally. This result is consistent with those obtained by Pal et al.^37^.

**Figure 4:**
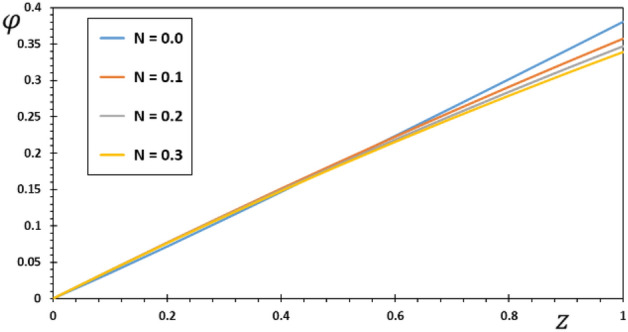
The electric potential $\varphi$ for various values of the gradient coefficient $N$.

With varied values of the gradient indicator $N$, the electric potential $\varphi$ vs. the distance $z$ can take on a wide range of shapes, as seen in Fig. 4. One can see that the electric potential quantities rise with increasing $z$ from the graph in Fig. 4. The highest value of the electric displacement is shown when the grading indicator declines. By comparing our findings to those published in^8,43^, we confirmed that they are consistent with previous research in the field. In the illustration, each curve starts with a value of zero, and they all satisfy the limit condition that $\varphi$ must be zero for $z$ to be zero.

**Figure 5:**
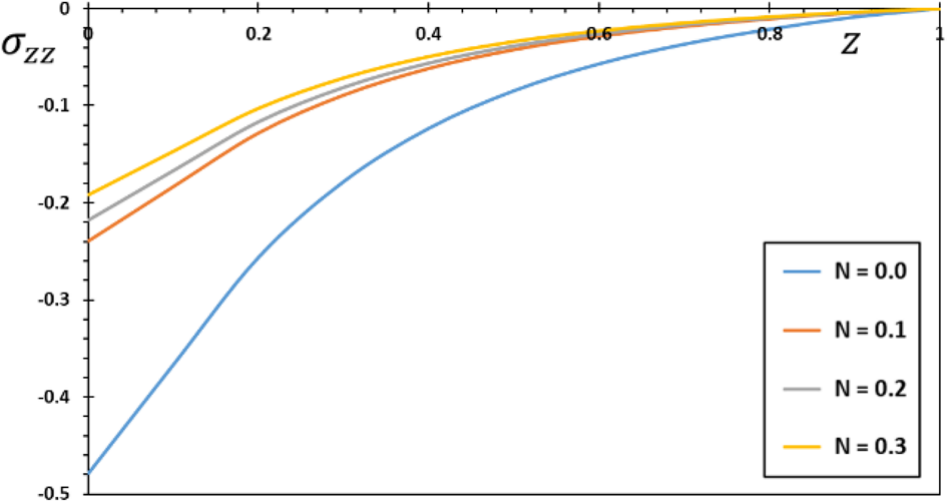
The normal stress $\sigma_{zz}$ for different values of the gradient coefficient $N$.

Figure 5 plots the change in thermal stress $\sigma_{zz}$ against the distance, z to see how the non-homogeneous parameter affects thermal stress $\sigma_{zz}$. When the space variable $z$ increases, the amplitude of the pressure $\sigma_{zz}$ in each case increases near the limit before decreasing. It is important to consider that these stresses express themselves as a result of the temperature change and the constraint posed by both ends of the $z$-axis extension. The highest points on the thermal stress distribution curve are moved from the end where the heat source was applied. Because the rod is clamped at both ends, thermal expansion displacement is prevented from developing along the length of the rod. As a result, piezoelectric stress develops in the piezoelectric FG rod. As seen in this figure, when the non-homogeneous indicator $N$ is modified, we also detect a significant variation in the levels of thermal stress $\sigma_{zz}$. The findings are consistent with those discussed in the sources^44,45^. At sufficiently high speeds and timeframes, the thermal behavior of the rod is also insensitive to the fluctuation in the speed of the heating source. The rod can be traced almost simultaneously when the source travels at extremely high speeds. Also, the same picture demonstrates that the thermal behavior of the rod reaches an asymptotic behavior at very high velocities.

The second scenario will investigate how memory-dependent parameters (time-delay factor ($\omega$) and kernel function $k\left( {t - \xi } \right)$) impact the behavior of all physical domains ($w$, $\theta$, $\varphi$, and $\sigma_{zz}$). It is important to remember that the kernel in the case of fractional differentiation is singular, whereas the kernel in the MDD framework is not. Also, at this point, the kernel can be considered more than just a memory manager. Many realistic models benefit more from the memory-dependent derivative concept than they would from the fractional-order derivative.

The kernel function is calculated using the formula (38). When comparing linear ($a = 0$) and quadratic ($a > 0$) functions for the kernel function, the focus of the comparison will be mostly on the influence of the kernel function on the structure of the interactions between the areas of work in both of those cases. The calculations have been carried out for each of the three distinct kernel functions using $K1 = 1$, $K2 = 1 - \frac{t - \xi }{\omega }$, and $K3 = \left( {1 - \frac{1}{\omega }\left( {t - \xi } \right)} \right)^{2}$.

Moreover, it is discovered that, depending on the time delay and kernel function choices, all distributions of temperature, displacement, stress, and electric potential disappear identically beyond a restricted region. However, the limited region is only time-related in the limited case of the modified piezoelectric thermoelasticity concept^32,46^, meaning that the thermal wavefront's position is continuously modified with time. The numerical findings show that the transient reactions to thermal and piezoelectric influences do not reach infinity instantly; that is, the thermal signal and the elastic wave travel at a limited velocity.

**Figure 6:**
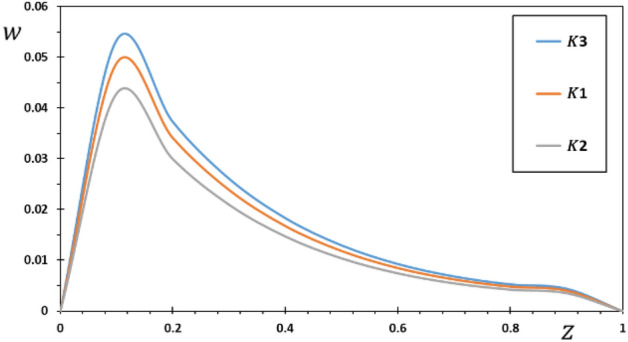
The displacement $w$ for various forms of kernel function $k\left( {t - \xi } \right)$.

**Figure 7:**
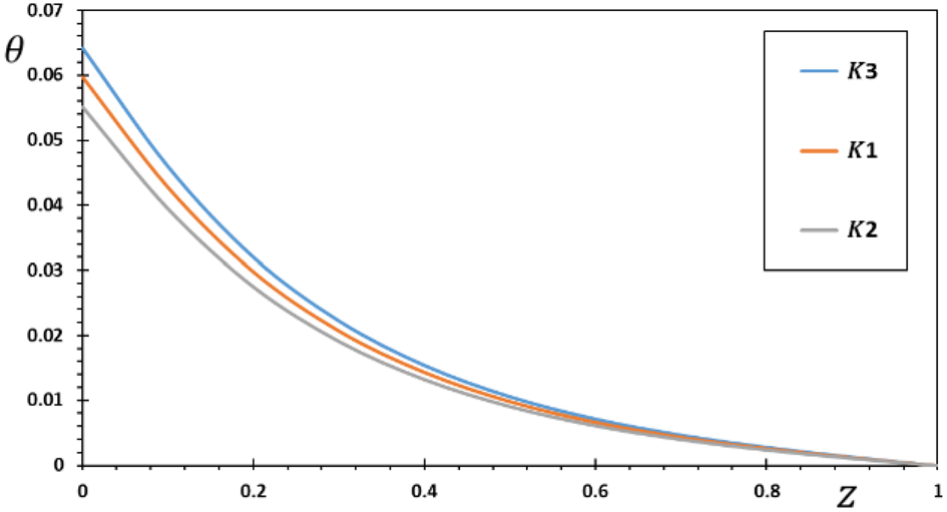
The temperature $\theta$ for various forms of kernel function $k\left( {t - \xi } \right)$.

**Figure 8:**
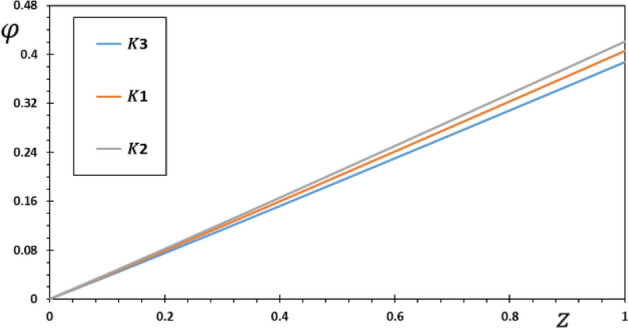
The electric potential $\varphi$ for different forms of kernel function $k\left( {t - \xi } \right)$.

**Figure 9:**
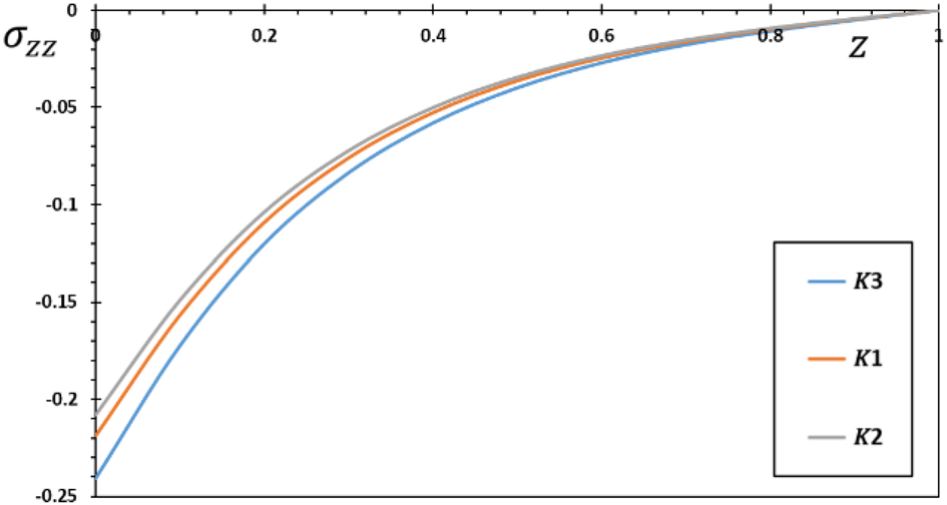
The thermal stress $\sigma_{zz}$ for different forms of kernel function $k\left( {t - \xi } \right)$.

The pattern of the curves shows how the kernel function influences the behavior of the rod, enabling verification of its goal. The studied fields were found to move more slowly than the memory effect would have predicted. Also, it is seen from the Figs. 6, 7, 8, 9 that the size of the different fields decays faster for kernel function $K2$ than for kernel function $K1$ or $K3$ values. The propagation of thermal waves is considerably influenced by time delay and kernel function: the inclusion of a memory-dependent derivative continuously smoothes and modifies the thermal wavefront; the greater the time delay, the quicker the thermal waves move. The outcome motivates further research into memory-dependent derivatives of thermoelastic materials as a novel field of practically relevant piezoelectric materials. Different transient reactions can be captured by selecting appropriate time-delay and kernel functions to meet the needs of certain applications. This means that there are now additional ways to describe the reactions and behavior of piezoelectric material in various settings.

## Conclusion

The current study explores the thermo-piezoelectrical behavior of functionally graded piezoelectric media (FGPM). The governing differential equations are generally found within the context of the generalized thermoelastic theory. Also, a modified Fourier's law was considered, including memory-dependent derivatives such as the nucleus function, time delay, and relaxation time. Based on this proposed model, the thermodynamic reaction produced by the moving heat supply of an FGPM thermal piezoelectric rod was investigated. The physical parameters of the FGPM rod are assumed to change significantly with length, except for environmental heat and thermal relaxation time. The analytical solutions of the governing partial differential equations have been found in the field of Laplace transformation. Numerical solutions can be obtained using a numerical Laplace inversion technique in the space–time domain. To clarify the discrepancies in the results between the generalized and coupled theories and the effect of different heterogeneity indices in addition to memory dependence, the numerical results of the transient response to FGPM are shown graphically. The following is a summary of the conclusions that can be drawn from the discussion of the numerical results:

- The change of the inhomogeneous index significantly influences the thermal stress behavior of the medium and the deformation propagation during uniform rotation of the rod.
- As the value of the heterogeneous index decreases, the solution becomes closer and closer to being the same as the solution to the homogeneous problem. For this reason, the effect of heterogeneity must be considered when designing delicate devices, such as smart materials.
- The presence of a moving heat supply on the considered medium significantly affects the magnitudes of the various thermophysical fields.
- The approach in this article can be generalized to solve some inhomogeneous thermoelasticity problems involving fast, short-range heat flow, such as lasers or moving sources.
- Since the kernel function can be selected randomly, the results show that the memory-dependent derivative is better for many real-world models than the fractional-order derivative.
- The responses of the physical fields moves slowly in the case of the existence of the memory effect by the evidence and physical experiments.
- The piezoelectric thermodynamic model derived in this work based on the memory effect can be useful when investigating the physical factors that most often determine the physical properties of particular materials. Also, the results may apply to various thermoplastic polymer and smart technology applications.

## Data Availability

All data generated or analysed during this study are included in this published article**.**

## Abbreviations

$${T}_{ij}$$: Stress tensor
$${S}_{ij}$$: Strain tensor
$${E}_{i}$$: Electric field
$${C}_{E}$$: Specific heat
$$\theta =T-{T}_{0}$$: Temperature increment
$${T}_{0}$$: Primary temperature
${\tau }_{0}$: Single delay time
$${u}_{i}$$: Displacement components
$${C}_{ijkl}$$: Elastic coefficients
$\varphi$: Electric potential function
${h}_{i}$: Heat flow vector
$$\rho$$: Density
$${p}_{i}$$: Pyroelectric quantities
$${\in }_{ij}$$: Dielectric parameters
${K}_{ij}$: Thermal conductivity coefficients
$${e}_{ijk}$$: Piezoelectric parameters
$${D}_{i}$$: Electric displacement
$$\eta$$: Entropy density
$$Q$$: Heat source
$${\beta }_{ij}$$: Thermal moduli
